# Influences on teleconsultation project utilization rates: the role of dominant logic

**DOI:** 10.1186/s12911-016-0392-2

**Published:** 2016-12-08

**Authors:** David L. Paul, Reuben R. McDaniel

**Affiliations:** 1 Department of Business Information and Analytics, Daniels College of Business, University of Denver, Denver, CO USA; 2 McCombs School of Business, The University of Texas at Austin, Austin, TX USA

**Keywords:** Teleconsultations, Telemedicine, Dominant logic, Complex adaptive systems

## Abstract

**Background:**

This research analyzes teleconsultation from both a mechanistic and complex adaptive system (CAS) dominant logic in order to further understand the influence of dominant logic on utilization rates of teleconsultation projects. In both dominant logics, the objective of teleconsultation projects is to increase access to and quality of healthcare delivery in a cost efficient manner. A mechanistic dominant logic perceives teleconsultation as closely resembling the traditional service delivery model, while a CAS dominant logic focuses on the system’s emergent behavior of learning resulting from the relationships and interactions of participating healthcare providers.

**Methods:**

Qualitative case studies of 17 teleconsultation projects that were part of four health sciences center (HSC) based telemedicine networks was utilized. Data were collected at two points in time approximately 10 years apart. Semi-structured interviews of 85 key informants (clinicians, administrators, and IT professionals) involved in teleconsultation projects were the primary data collection method.

**Results:**

The findings indicated that the emergent behavior of effective and sustainable teleconsultation projects differed significantly from what was anticipated in a mechanistic dominant logic. Teleconsultation projects whose emergent behavior focused on continuous learning enabled remote site generalists to manage and treat more complex cases and healthcare problems on their own without having to refer to HSC specialists for assistance. In teleconsultation projects that continued to be effectively utilized, participant roles evolved and were expanded. Further, technology requirements for teleconsultation projects whose emergent behavior was learning did not need to be terribly sophisticated.

**Conclusions:**

When a teleconsultation project is designed with a mechanistic dominant logic, it is less likely to be sustained, whereas a teleconsultation project designed with a CAS dominant logic is more likely to be sustained. Consistent with a CAS dominant logic, teleconsultation projects that continued to be utilized involved participants taking on new roles and continuously learning. This continuous learning enabled remote site generalists to better handle the constantly changing nature of the problems faced. A CAS dominant logic provides a theoretical framework which explains why the teleconsultation literature about the role of technology, which is based on a mechanistic dominate logic, does not have adequate explanatory power.

**Electronic supplementary material:**

The online version of this article (doi:10.1186/s12911-016-0392-2) contains supplementary material, which is available to authorized users.

## Background

Early utilization rates of installed teleconsultation projects in the 1990s were disappointingly low [1, 2] despite being perceived as an effective means to address many of the challenges of healthcare delivery access and quality issues in medically underserved areas [1, 3, 4]. Teleconsultations are consultations between two or more geographically separated healthcare providers connected through information and communications technologies to provide value-added healthcare delivery [1, 4, 5]. The participants generally include a primary care provider (family practice physician, nurse practitioner, or physician assistant) located at a local hospital or clinic and the relevant specialist(s) located at a university-affiliated health sciences center. The patient may or may not be present.

Teleconsultation technologies vary by project and network. Many teleconsultation projects utilize commercial videoconferencing equipment combined with HIPAA-compliant encryption hardware or software. Specialized teleconsultation workstations that also include some combination of additional cameras, light sources, and the ability to connect medical devices such as endoscopes and otoscopes were also utilized. Like other information and communication technologies, the capabilities, usability, and affordability of teleconsultation technology have significantly increased over time [6].

Numerous studies have demonstrated the efficacy and efficiency of many types of clinical applications in teleconsultations in general [7–10]. These include but are not limited to: neurology [11], coronary artery disease, [12], dermatology [13], inflammatory bowel disease [14], military-related post-traumatic stress disorder [15], oncology [16, 17], and various aspects of managing diabetes [18, 19]. Further, numerous teleconsultation projects have been successfully implemented and studied [20–22], and significant financial support has been given to projects targeting medically underserved areas such as rural America [23, 24]. Yet teleconsultation project utilization rates have remained low [25, 26], and, few projects effectively implemented have been sustained over time despite a continued need for services [20, 22, 25].

The main causes generally accepted for teleconsultation not becoming more widespread include technology issues, uncertainty about licensure, legal liability, and, most importantly, reimbursement [1, 26]. However, empirical support for these explanations is almost nonexistent. Yet these beliefs continue to dominate discussions about the inability of teleconsultation to become more widespread.

One reason for this may be that the dominant logic which traditionally drives the design of teleconsultation projects has been problematic. Dominant logic, developed in the business strategic management field, “consists of the mental maps developed through experience in the core business and sometimes applied inappropriately in other businesses” ([27 p. 485), where mental maps are explicit cognitive maps that facilitate the learning and recall of information and the construction and accumulation of knowledge in a manner that reduces an individual’s cognitive load and enables them to makes sense of and plan activities for a given situation [28, 29]. Teleconsultation projects traditionally have been evaluated based on a mechanistic dominant logic, where the dominant logic is how teleconsultation is similar to traditional service delivery systems. However, teleconsultation may be fundamentally different from traditional service delivery systems and therefore need to be evaluated utilizing a different perspective. This paper argues that a teleconsultation project is a complex adaptive system (CAS) and that a CAS dominant logic must be utilized to understand them. A CAS dominant logic examines emergent behavior resulting from interactions and relationships between system agents. The purpose of this research is to better understand how a complex adaptive system dominant logic of teleconsultation differs from a mechanistic dominant logic of teleconsultation. We also identify the implications for researchers and practitioners of applying a CAS dominant logic to evaluation and understanding of teleconsultation projects.

### The Dominant Logic of Teleconsultation Projects

Dominant logic is a linkage between organizational performance and environmental driven organizational change in that it predisposes organizations to certain kinds of strategic problems and often interacts with organizational systems and structures in a complex way. It is especially relied on in situations that are information rich but interpretation poor Dominant logic is an information filter which influences where organizational attention is focused, and this puts constraints on the ability of the organization to learn and unlearn, especially the longer it has been in place [30].

#### A Mechanistic Dominant Logic of Teleconsultation

A mechanistic dominant logic perceives teleconsultation as similar to those service delivery systems where work processes are carefully spelled out and roles are assumed to be fixed. Performance under these conditions is a function of individuals knowing their roles and executing them, and poor performance is the result of people not knowing their roles or not executing them properly. With a mechanistic dominant logic, actual system behavior is expected to be the same as planned or intended behavior, and any deviation between the two is perceived as a negative.

Teleconsultation projects initially designed with a mechanistic dominant logic are based on the traditional service delivery model where primary care providers transfer responsibility of their difficult patients to specialists at another location. Each provider would have a clearly understood role and would be expected to behave in that role. Thus, understanding causes of low utilization rates of implemented teleconsultation projects would focus on the extent to which teleconsultation sessions differ from face-to-face sessions. This may help explain why insufficient technology capabilities, reimbursement, licensure, or legal liability concerns are accepted as the reasons, despite almost no empirical support, why teleconsultation have not become more widespread. It may be that a different dominant logic is needed to better understand why teleconsultation projects have not come close to reaching their potential.

#### A Complex Adaptive System Dominant Logic of Teleconsultation

CASs, drawn from the field of complexity science [31–33], are qualitatively different from linear systems often studied in more traditional sciences. In CAS, system behavior is emergent and the collective result of nonlinear interactions [34] among diverse agents [35, 36]. Agents are information processors [32, 33, 37–39] that can adjust their own behavior and learn as a function of information they process [31] through their interactions among each other and with their environment.

Critical to the concept of CASs is that system behavior is emergent and nonlinear [32, 38, 40], and is the product of coupled, context-dependent interactions and relationships between diverse, independent agents. Relationships are critical; however, agent diversity plays a role because it is a source of novelty and adaptability, and it is by diverse agents interacting with each other that the system is capable of learning [38]. Therefore, understanding a CAS requires understanding patterns of relationships among agents rather than simply understanding the nature of individual agents.

The dynamic, emergent behaviors resulting from nonlinear agent interactions makes these systems qualitatively different from static systems that may be complicated, but are not complex. Being emergent also means that system behaviors are fundamentally unknowable in their trajectories [31–33] because overall system behavior cannot be obtained by aggregating the behaviors of the constituent parts [32]. Rather, new patterns of relationships among agents manifest themselves, and these patterns of behavior are discernable and can be studied. As such, understanding CAS behavior and characteristics is the result of detecting and understanding these patterns [32, 33, 36].

Teleconsultation projects are CAS because they have multiple end-users (agents) with different skills, knowledge, and expertise working together to accomplish goals in an emergent fashion. A CAS dominant logic causes one to attend to the emergent behavior of the system resulting from the relationships between the agents. It assumes that agents (in this case, the healthcare professionals participating in the teleconsultation projects) learn and roles are flexible. Performance is based in part on the ability of agents, and the relationships between agents, to change or evolve and adapt over time. Each agent interacts with other agents and learns and assumes new roles or takes on additional responsibilities as he or she does so. Poor performance is the result of agents not learning and evolving, or the relationships between agents becoming stagnant over time.

## Methods

### Research Design

Qualitative case studies of 17 teleconsultation projects from four telemedicine networks (Sites W, X, Y and Z) were included in this research. Data from telemedicine networks W, X, and Y were collected at two points in time nearly a decade apart while data from Site Z were collected at the later data collection period only. Sites W and X did not have any active teleconsultation projects at the time of the second data collection period. The Institutional Review Board (IRB) of the university approved these projects.

### Sample

Telemedicine networks, consisting of a university-affiliated health sciences center (HSC) as the hub and smaller healthcare facilities such as physician offices, clinics, and non-tertiary care hospitals as the spokes, were purposely selected because the vast majority of civilian teleconsultation projects at the time of this research involved HSCs [41]. HSC teleconsultation projects tended to have certain characteristics that naturally account for alternative explanations of installed project utilization [42]. Three networks (Sites W, X, and Y) were initially studied in the first data collection period.

The research project during the first data collection period involved not only teleconsultation but also teleradiology and distance learning telemedicine projects. Site selection was based on four criteria. First, each site had to have at least three active telemedicine projects. Second, each site had to have one of each of the three types of telemedicine activities: teleconsultation, distance learning, and teleradiology. These two criteria enabled both within and between network comparisons of different telemedicine projects. Third, the sites could not involve military or correction facilities because the voluntariness of participation and the dynamics of trust in such situations may be different from those in civilian projects. Fourth, each site had to have been operational for a minimum of 6 months to allow the inevitable technological and procedural bugs to be addressed and to allow the novelty of telemedicine to pass.^1^

The World Wide Web was searched to find sites that met these criteria. The second criterion—different types of telemedicine activities within each project—was discarded because sites meeting this criterion could not be found. Although a number of potential sites claimed to have all three types of telemedicine activities operational at the time of the first data collection period, only one actually did. Indeed, a number of potential sites that claimed on their Web pages to have active telemedicine projects did not have any active telemedicine projects at that time. This exaggeration of the state of active telemedicine projects was not uncommon. The ORHP [2] found that approximately 25% of the hospitals they surveyed which claimed to have at least one active telemedicine project in fact had no operational telemedicine projects. Each site selected included at least one teleconsultation project, which enabled teleconsultation activities to be compared across the telemedicine networks. The researchers did not have specific types of teleconsultation projects in mind for their research and thus included any operational teleconsultation projects that were available during the data collection periods. The researchers argue that the variability in the types and locations of the teleconsultation projects and the participants themselves helped strengthen generalizability of the findings.

Additional file 1: Table S1 presents background and demographic information about the projects. A total of 17 teleconsultation projects in 14 geographical locations were studied (three remote areas each had two different teleconsultation projects located in the area). All the telemedicine networks and their teleconsultation projects were located in the Southwestern United States. All of the remote sites were designated as either medically underserved areas or populations, and 15 of the 17 remote sites were designated as primary care Health Professional Shortage Areas (HPSAs). The two remote sites not designated HSPAs, Z2 and Z3, were located in the same relatively isolated city and surrounded by areas within the county that were designated HPSAs.

Population size of the remote sites varied. Twelve of the 17 remote sites were in areas located in US Department of Health and Human Services designated non-metropolitan (population less than 50,000), with the rest being defined as metropolitan (population over 50,000). The ratio of sites studied that were classified as metropolitan or nonmetropolitan (29%/71%) is consistent with the United States as a whole (27%/73%) [43]. Each remote site was relatively isolated geographically, with the nearest HSC being a minimum of 60 miles away. Twelve of the 17 projects remote sites were 200 or more miles from the nearest HSC.

Data were collected from five teleconsultation projects during the first data collection period. As indicated in Additional file 1: Table S1, Site W had an oncology—bone marrow transplant teleconsultation project that was used for both initial patient screening and posttreatment follow-up where the patient was usually present. Site X had a pediatric oncology teleconsultation project used for follow-up where the patient was always present, and a multiple drug-resistant tuberculosis teleconsultation project used primarily in conjunction with inpatient treatment, where the patient was rarely present. Site Y had two multiple medical specialties teleconsultation projects which involved the diagnosis, treatment, and follow-up of numerous medical conditions which a primary care provider usually would refer patients to a specialist. Whether or not the patient was present during the teleconsultation session was primarily dependent on the medical condition and the usefulness of the patient’s presence.

The researchers planned to revisit these sites in order to study how these teleconsultation projects had changed over time. The second data collection period occurred approximately a decade later and focused solely on teleconsultation projects. A status update for Site W was received by their former Associate Director of Telemedicine, and Sites X and Y were revisited. Data about the status of the operational teleconsultation projects at the first data collection period and additional teleconsultation projects implemented since then were collected. Unfortunately, both Sites W and X had decided to discontinue or deemphasize their teleconsultation efforts. Site X had decided to focus on distance learning only, and their tuberculosis teleconsultation project had been transferred to a different HSC that was not part of this study (the infectious diseases specialist formerly affiliated with HSC X transferred her affiliation to that different HSC but remained in the same location as before). Site W also had significantly deemphasized their teleconsultation efforts because state funding for the HSC as a whole had been significantly reduced, and their teleconsultation projects were one of many efforts whose funding was cut. Site W had some efforts involving neonatal CT-scans readings and telepsychiatry serving Native American populations but the researchers were unable to secure access to these. For both HSCs, the decisions to discontinue or deemphasize their teleconsultation efforts were made at the organizational level.

During the second data collection period, Site Y had five active teleconsultation projects. This included one multiple medical specialties teleconsultation project from the first data collection period (the other had been discontinued), and four additional teleconsultation projects that had been initiated since that time. The burn unit teleconsultation project was used primarily for long-term follow-up and treatment after the patient had been released from the hospital, and the patient was always present. The oncology teleconsultation project was used for the administering of chemotherapy, where the patient was always present, and addressing related side effects, where the patient sometimes was present. The primary care teleconsultation project involved a remote site primary care physician linked to an even smaller town which also had a telepharmacy link with the HSC. The health care provider at the smaller town was the local emergency medical technician and the patient was always present. The pediatric care teleconsultation project was located at the rural site’s elementary school where the patient was present on an as-needed basis.

In addition, data were collected from a fourth telemedicine network, Site Z, during the second data collection period. Data about Site Z were not initially collected because, at that time, they did not have any active teleconsultation projects. Eight teleconsultation projects involving three different clinical applications were studied at Site Z. Project HCV had four teleconsultation projects focused primarily on hepatitis C, including determining whether a patient was a good candidate for treatment. It was also used for the management of both the disease itself and treatment side effects. The patient was never present. Project ECDD had three teleconsultation projects dealing with early childhood developmental disabilities where the patient was always present during diagnosis and treatment, but not during training sessions. Project DABC had one teleconsultation project dealing with drug abuse and behavioral counseling where the patient was not present for the case discussions but occasionally present when needed. With the exception of the burn unit teleconsultation project at Site Y, none of the remote sites had any type of formal affiliation or reporting relationship with the HSCs with whom they partnered.

#### Teleconsultation Technology

As presented in Additional file 1: Table S1, all six of the teleconsultation projects studied at Site Y both during the first and second data collection periods utilized a basic modular teleconsultation workstation which HSC Y had designed and later licensed the manufacturing of to a major Japanese electronics company, because the specialty teleconsultation equipment available at the time was perceived as being both too complex and too costly for their requirements. Their teleconsultation workstation was put together with off-the-shelf components and included a full motion video codec (coder/decoder), an x-ray light box, and a one chip CCD camera which could be used to view the patient or tilted downward to view x-rays or documents. The workstation also included a video examination camera with a universal adapter to fit endoscopic applications, a high-powered xenon light source for general lighting purposes or for direct application to endoscopic devices, and an otoscope which could be directly attached to the exam camera and xenon light source. A unidirectional microphone was attached to the unit, and on top of the cabinet were two small high-resolution monitors, the larger showing the image being transmitted, and the smaller one showing the return transmission signal. A VCR was available to record and document teleconsultation sessions. The unit also had additional data ports and auxiliary audio/video inputs and outputs. During the second data collection period, a number of Site Y remote sites, including Y2 (medical specialties) and Y6 (school clinic), still utilized upgraded versions of that same workstation. The other Site Y remote sites utilized a newer generation of their basic teleconsultation workstation.

Nine of the remaining 11 projects studied utilized some variation of commercial off-the-shelf videoconferencing equipment (although multiple drug resistant tuberculosis teleconsultation Project X2 switched back to telephone, email, and facsimile during the second data collection period). At Site Z, no standard teleconsultation workstation was deployed throughout its network. Six of the eight teleconsultation projects studied at Site Z utilized videoconferencing equipment. The two other teleconsultation projects (Project HCV Z1 and Z3) utilized teleconferencing, although both were planning on migrating to videoconferencing in the near future.

At Site Z, both Projects HCV and DABC deployed basic Polycom videoconferencing equipment because the nature of their teleconsultation sessions required very limited technology capabilities. Such sessions generally involved a discussion between the various healthcare providers, although Project DABC sometimes included a patient being present. Project ECDD presented more difficult challenges from a technology perspective in that they had multiple, different teleconsultation workstation configurations and often used other project’s teleconsultation workstations as well. Further, as was standard practice in their field, Project ECDD also required equipment that could be used at the patient’s home. At the time of the second data collection period, they were on their fourth generation of teleconsultation workstations and had begun purchasing standard laptops equipped with HIPAA-compliant encryption software.

The teleconsultation projects studied in the first data collection period all utilized dedicated point-to-point telecommunication links—primarily because this was the only option available. These telecommunication links, usually either T1 lines or satellites, were very expensive (up to $3500 per month–although a number of states subsidized the cost) and thus unsustainable in the long run. At the time of the second data collection period, all the teleconsultation projects studied that were not utilizing teleconferencing as their main communication link were now using IP-based multipoint telecommunication networks. All of Site Y’s teleconsultation projects connected to the same educational and healthcare-related designated IP-based multipoint telecommunication network that had been implemented throughout the state. For Site Z, a statewide telecommunication network had not yet been fully deployed, and different teleconsultation projects utilized different telecommunication networks, or some combination thereof, to provide the connections between the HSC and the remote sites. These included a state-based educational network and networks belonging to different federal agencies.

### Data Collection

Data were collected at two points in time (1996/1997 and 2007) approximately 10 years apart, and the primary data collection method involved face-to-face, issue-focused, semi-structured interviews of key informants (sample interview questions are available in the Appendix). The time elapsed between the two data collection periods was based on a desire to be sure that projects had been in existence long enough to become institutionalized in the delivery setting. Face-to-face interviews were required to collect the thick and richly textured data that were needed to understand the topics being researched [44–46] because, prior to the first data collection period, telephone interviews were pretested and found ineffective.

Table 1 presents an overview of the distribution of key informants, who were members of one of three groups—clinicians (physicians, physician assistants, nurse practitioners, medical residents, nurses, or, in one case, an emergency medical technician), administrators, and IT professionals. They were selected based on current or past direct involvement in their organization’s teleconsultation projects. A total of 85 healthcare professionals, 8 at Site W, 17 at Site X, 35 at Site Y, and 25 at Site Z, were interviewed face-to-face, and the interviews were audio recorded and transcribed. At Site Y, 17 were interviewed during the first data collection period, whereas 21 (including three from the first period) were interviewed during the second data collection period. At Site X, 15 were interviewed during the first data collection period and five (including three from the first period) during the second, while at Site W, eight were interviewed during the first period and one was reinterviewed during the second data collection period.

**Table 1:**
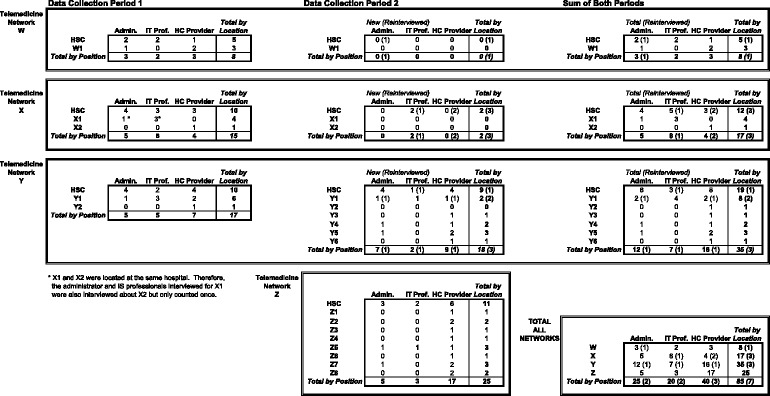
Key informants overview

Both data source and method triangulation [47, 48] were utilized in an effort to improve validity and reliability [49–51], and partially address key informant bias issues [49, 51]. Data source triangulation was accomplished by interviewing at different times multiple key informants from the three different functional groups at both the remote healthcare facility (if multiple key informants existed) and the HSC involved in each teleconsultation project studied. Although semi-structured interviews of key informants were the primary data collection method, within-method triangulation [47, 48] was also utilized. As indicated in Additional file 1: Table S1, this varied by teleconsultation project but included observing teleconsultation sessions or video recordings of such sessions and analyzing documentation such as grant proposals/follow-up, needs assessments, and strategic plans. This was done in an effort to verify factual data and corroborate key informant answers. However, there were cases when such data were not documented or privacy issues prevented a researcher from having access to it. In these cases, data collected from multiple key informants were used to corroborate the answers.

### Data Analysis

The transcribed interviews were analyzed and coded after each data collection period in which they were collected, based on the coding scheme presented in Table 2. The coding scheme was developed prior to the first data collection period based on the relevant literature and was fine-tuned over time. Interviews relevant to a particular case (teleconsultation project) were first coded, and the coded interview segments for that given case were grouped together, analyzed, compared, and integrated in an iterative process. Each case was written up on its own in order to integrate the relevant interviews for each teleconsultation project into one document. This resulted in a more complete and coherent understanding of each individual project than would have been possible by analyzing each interview separately. During the case write-ups, it occurred to the researchers that the application of a CAS dominant logic might help shed light on what had been observed. Each case was then reanalyzed and compared against the others using both dominant logic frameworks. The use of computer-aided qualitative data analysis software enhanced coding reliability by making possible more consistent, frequent, and in-depth comparative analysis [52–54]. It also enhanced confidence in internal validity by more readily facilitating the constant comparison and pattern matching of the different coding values assigned first within and then between cases [52, 54, 55].

**Table 2:** Coding scheme overview

I. GENERAL PROJECT BACKGROUND/PARTICIPANTS	VI. TELCONSULTATION SESSIONS
A. HSC Specialty	A. TC Session Scheduling
B. Physical/Plant Description of Remote Site	TC Frequency
Remote Site Resource Issues	TC Session Length
	B. TC Session Description
II. HEALTH CARE DELIVERY PROBLEM	TC Session Process
A. Health Care Complexity	
Diagnosis	VII. PROJECT IMPACT
Treatment	A. TC Outcomes -- Examples
Disease Quirks	TC Outcomes (Before/After)
	TC Outcomes -- Failures
III. PROJECT INITIATION (when/why started)	TC Outcomes -- Readmittance
A. Date Project Started	B. Access to Care -- Overall
B. Project Startup	Access to Care -- Project Volume
Initial Activities	Access to Care -- TC Efficiency
Barriers to Startup	C. Cost of Care -- Overall
C. Need for Project	Cost of Care -- Project Financing
	Cost of Care -- Reimbursement
IV. INFORMATION TECHNOLOGY (IT) CONFIGURATION	D. Quality of Care -- Overall
A, IT Equipment	Quality of Care -- Reputation
IT Location	Quality of Care -- Referral Patterns
IT Description	Quality of Care -- Local Expertise
B. User Perceptions	
IT Training	VIII. PROJECT FUTURE
IT Ease-Of-Use	A. Future -- Issues to Be Resolved
IT Reliability/Problems	
IT Sufficiency	IX. DOMINANT LOGIC (2nd data collection period only)
Local IT Support Availability/Expertise	A. Intended Versus Actual Behavior
	B. Participant Roles and How They Changed
V. TELCONSULTATION (TC) PROJECT BACKGROUND	C. Impact of Teleconsultation Projects Where
A. TC Project Purpose	Actual Behavior Was Similar to Intended Behavior
B. Patient Demographics	Actual Behavior Was Emergent Behavior of Learning
C. TC Participants	*Types of Learning*
TC Participants -- HSC	
TC Participants -- Remote Site	
*Remote Site Participant Expertise*	
*Remote Site Training*	

The coding for both data collection periods was performed by the first author. For the first data collection period, the coding of variables for each teleconsultation project that could not be directly assessed were assessed by another information systems researcher. The third party assessor concurred with the researcher’s coding 94% of the time. The coding for the second data collection period was reviewed by the second author but was not formally assessed because the volume of interviews and the lack of funding made it impractical to seek other coders. It is argued that the reliability and validity of the coding is sufficient because the coding related to this manuscript primarily involved identifying factual information and not subjective judgments by the coder, and the coding scheme used for both data collections periods was similar and performed by the same researcher. Third party assessment of the coding of the data from the first data collection period indicated that the coding was reliable and valid, and there are no reasons to believe that the same does not hold for the second data collection period.

While researcher triangulation [47, 48] was limited in the coding process, researcher and theory triangulation [47, 48] in terms of interpreting the findings were important drivers for this article. The motivation for this research was that what was observed in the data collection periods was not consistent with the generally accepted framework for the role of technology in teleconsultations. The second researcher suggested applying a complex adaptive systems perspective to better understand technology’s role.

As previously stated, key informant interviews were the primary data collection method. Note that in the Results section examples usually involve only one key informant; however, whenever available (including those presented), multiple confirming comments from different key informants involved in that particular project, in addition to other forms of evidence, were used to determine the findings. Further, key informant quotes from the first data collection period are specifically identified, with the others all being from the second data collection period.

## Results

Teleconsultation projects were analyzed from both a mechanistic and complex adaptive system dominant logic in order to further understand the influence of dominant logic on utilization rates of teleconsultation projects. The presentation of the results is organized by theme across the different cases rather than presenting each case individually in order to emphasize that the findings generalized across cases. The findings indicate that in teleconsultation projects that were sustained over time, actual utilization tended to differ from the intended behavior in that it involved learning, and that this continued utilization required a change in and the expansion of participants’ roles. Such learning—especially if it was continuous—had a positive impact on remote site health care delivery in that it enabled remote site healthcare professionals (RSHCPs) to manage and handle a greater variety of and more complex cases on their own.

### Teleconsultation Project Utilization

#### Intended Versus Actual Behavior

The findings indicated that the teleconsultation sessions’ primary use very rarely was based on a traditional service delivery model where the RSHCPs would hand off the responsibility of their patients with difficult cases to HSC specialists, nor were they used for definitive diagnosis by the HSC specialists. This was because the RSHCPs were instead looking for guidance and help in making sense of the situations they faced. A remote site physician involved with multiple specialties teleconsultation project Y1 during the first data collection period commented:

*You know, most of us can figure out what needs to have a procedure and which ones don’t. Most of its coming down to, you know, data management, reassurance, and that kind of thing. Very rarely do we not have any kind of idea at all of what is happening.*

None of the projects that were significantly utilized or sustained primarily involved the RSHCPs handing off responsibility for their patients to the HSC specialists. Indeed, the projects that attempted to engage in the planned behavior only (X1 pediatric oncology and Y2 multiple medical specialties) were either quickly discontinued or for all practical purposes inactive. Instead, the behavior of those projects that were sustained was emergent and focused on facilitating learning by the participating healthcare professionals. HSC specialists involved in the projects that had a positive impact on remote site healthcare delivery realized this and consciously and deliberately focused on relationships in the projects being used for learning in order to empower the RSHCPs to handle more healthcare problems on their own. For example, Project ECDD’s specialists did not want to be perceived by the remote sites’ developmental specialists as experts to whom difficult cases could be transferred. Therefore, the teleconsultation project focused on empowering remote site developmental specialists. Project ECDD’s Senior Communication Specialist explained:

*I am trying something a little bit different because I do not want to be perceived as the expert on the other side of the TV set telling these people what to do…(W)e are really working to empower the service coordinators and the families to implement things within their everyday life, and that it is not a magic hands therapy technique to change these kids.*

Project ECDD’s Program Consultant expanded on this idea:

*(T)hat’s the philosophy behind it (teleconsultation project) is to really build on their (remote site developmental specialists’) strengths that they already have and to show them that they’re really on the right track, and we’re just going to offer you little pointers along the way to enhance that. “You guys (RSHCPs) know a lot. You need to know that you do have the skills to do more of what you’re thinking that you can’t do, and that’s why we want to support you,” as opposed to assuming that you need a therapist or a specialist to come in and do this the right way or do this because you can’t do it at all.*

#### Continued Utilization Required Change in and Expansion of Participants’ Roles

The findings indicated that in teleconsultation projects that continued to be effectively utilized, the roles of the participants evolved and were expanded. HSC specialists had to change their role from that of information provider and decision-maker to one of teacher or coach, while RCHCPs had to evolve from instruction takers to learners and thinking decision-makers. For HSC specialists, having the requisite expertise was a necessary but not sufficient condition for learning emerging as the primary system behavior. Equally important was that HSC specialists had the personality and interpersonal skills necessary to create an effective learning environment. If the HSC specialist was not willing or able to take on the role of teacher, then evolution of the system to one facilitating learning was difficult. Z4’s PA gave an example of how the HSC hepatitis C specialist had embraced the role of teacher:

*(H)e (HSC HPV Specialist) makes the point to make every opportunity to learn (that) is possible. If somebody presents a patient and there’s any opportunity, he’ll say, “Okay, let’s stop here. I wanna explain why I’m gonna tell you to do this.” And he’ll explain it and you leave feeling like I totally know this now.*

RSHCPs also had to have the willingness and ability to learn and assume new roles. Project ECDD’s Senior Program Therapist gave an example of how both his role and the role of the developmental specialists changed. In this case he was coaching them by actively teaching them how to more effectively work with young children. He stated:

*(W)hat you have to do with the little kids is crawl on the floor, play with what they’re interested in, teach through playing. So in some ways it’s very different from what they’re (RSHCPs) used to doing. And so you’re helping them learn how to teach little kids in a different way.*

The burn unit teleconsultation project was an excellent example where the roles that both the HSC specialist and the RSHCPs played changed significantly and, as a result, their relationship changed significantly as well. In this case, the HSC specialist made a concerted effort to teach the RSHCPs not only what needed to be done but also why they were doing it and what they were looking for. In other words, the RSHCPs became learners, thinkers, and decision-makers. This resulted in the RSHCPs having expanded expertise that enabled them to both better predict what the HSC specialist would need and to proactively act without being asked. This often resulted in the RSHCP taking on a number of additional roles and responsibilities not necessary in a face-to-face setting. For example, in the burn unit teleconsultation project, Y3’s Nurse had become so familiar with what HSC Y’s Burn Specialist wanted that he no longer had to ask her to do things to assist his assessment. Instead, she understood what he was trying to accomplish and now did the necessary steps proactively. YM’s burn unit specialist explained:

*(T)hey (Y3 Nurse) know why I would be holding it (the patient’s arm)…So it used to be I’d say, “Okay, (Y3 Nurse), do you feel anything, grinding, popping?” Now it’s just, “And doctor I don’t feel any popping or grinding.” It’s just all second nature to them now. So it just kind of happens with that, that’s all.*

#### Impact of Teleconsultation Projects with Emergent Behavior of Learning

This emergent behavior of learning enabled RSHCPs to manage and treat more complex cases and healthcare problems on their own without having to refer to the HSC for assistance. As the head of HSC Z’s hepatitis C teleconsultation project explained:

*And by using case based learning, then they will rapidly become experts in these diseases. And that’s what you probably saw, how they were becoming better and better, you know? They were all listening to each other, and so they were learning rapidly. And what we have shown is they rapidly become experts so that we can enhance capacity to care for these common problems and that’s the whole idea here…we can set up these knowledge networks and train their own people to manage their own patients.*

An example of the impact projects whose emergent behavior focused on facilitating learning had was demonstrated by RSHCPs description of how their expertise and competency had increased. Z4’s PA believed participating in Project HCV enabled her to handle more complicated and complex problems on her own:

*Interviewer: Has your definition of a complication remained stable over time?*

*PA: <chuckles > It’s changed, yeah. Already, it’s changed a little bit because in the beginning, I felt so inexperienced with it that any change was a complication to me. I was scared to death.*

The evidence suggested that this increased expertise had tangible benefits in terms of remote site healthcare delivery. Project HCV had collected preliminary data showing that the outcomes for patients treated for hepatitis C via the teleconsultation project were as good if not better than the results of patients being treated at HSC Z only. Z4’s PA commented:

*And that’s what [Project HCV Specialist] is trying to closely monitor, is are our outcomes in these rural communities the same as his in the big university using the teleconferencing? And they are. They’ve done studies that show—as a matter of fact, they might be better.*

In the case of the tuberculosis teleconsultation project at X2, the teleconsultation project enabled the remote site to treat even more complicated cases of tuberculosis at a lower cost. A physician at X2 noted during the first data collection period:

*It’s [teleconsultations] become our workhorse—especially for infectious diseases… People talk about cost-effectiveness. One case of infectious diseases costs the state $250,000. Since we introduced telemedicine, we’ve gotten that down to less than $100,000. So you figure then—you know it’s one thing when you have ten of those cases. Now when you look at 184 or 289 or 500 and it grows exponentially like that.*

#### Types of Learning

Within a teleconsultation project, some types of learning that emerged had a more significant impact on remote site healthcare delivery than did others. Projects that focused on information transfer, where the HSC specialist conveyed his or her expertise to the RSHCP in a more sequential manner did have a positive impact on remote site healthcare delivery. The former Director of Telemedicine at HSC Y explained how during the first data collection period:

*(T)he presenter (RSHCP) is the biggest benefactor of all this because, after awhile, he becomes damn sharp talking to the chair of orthopedics 1 h a week. After a while, well, why in the hell am I going to present that case again? I already know how to handle it. So the education that goes on for the presenter, whoever he is, at whatever category, is absolutely, you become super at what you do.*

However, this type of information exchange-based learning also tended to result in the reduction in the number of teleconsultation sessions. The former Director of Telemedicine at HSC Y continued:

*And, therefore, they use the teleconsultative services is like this, whew! And then it (teleconsultation project utilization) goes down, well why? Because I can handle a hell of a lot. How much could they handle that they could not handle before? I’d say 30%!*

In contrast, teleconsultation projects whose emergent behavior involved continuous learning tended to have a bigger and more sustained impact on remote site healthcare delivery. The nature of the healthcare problems faced by RSHCPs was constantly changing, and continuous learning enabled them to expand their expertise and thus their ability to handle more problems on their own on a regular basis. This appeared to hold regardless of whether RSHCPs were physicians, physician assistants, nurses, therapists, or developmental specialists. The Nurse at Project HCV Site Z2 stated, *“With every presentation and every patient you have, you’re constantly learning. You’re constantly learning.”* This belief was echoed by Project HCV Site Z1’s Physician when he stated:

*And the whole idea is that you learn quite a bit. If maybe ten people call in, and everybody is presenting a patient. By listening, I learn of a patient’s problem and what to do about it, you know?…So it’s like a continuous wheel for learning, you know what I mean?*

As a result, teleconsultation projects whose behavior attempted to address medically underserved healthcare delivery challenges by transferring the responsibility for patients with more challenging healthcare issues to HSC specialists were less likely to be sustained over time. In contrast, teleconsultation projects whose emergent behavior focused on continuous learning enabled RSHCPs to address a greater variety and more difficult cases locally. A RSHCP affiliated with HSC X’s tuberculosis project explained during the first data collection period:

*One of the items against telemedicine is well…I mean once I learn how to treat TB, I don’t need it anymore and what’s the fun of having it. And what we found is we’re taking care of more cases and more complicated cases and the number of attendees to the (tele)conferences has actually gone up. Normally the studies that I’ve seen on telemedicine, they’ll show this initial peak and then it drops off as interest goes. The number of cases that we’re presenting are obviously going way up.*

### Role of Technology

#### Technology Not A Cause of Project Curtailment or Discontinuation

None of the teleconsultation projects studied in either the first or second data collection period were curtailed or discontinued because of technology issues. Further, as discussed below, when technology issues were mentioned, the problems were not related to the capabilities of the technology being insufficient, but rather the technology being too sophisticated or the users not having enough training to utilize the capabilities available. Instead, the findings indicated that the technology required for teleconsultation projects whose emergent behavior was learning did not need to be terribly sophisticated. The physician involved with Project Y5 explained:

*You know, the technology, for the most part, is window dressing, I think. Now, in terms of being able to safely take care of someone, you don’t really need a whole lot of technology.*

#### Technology Needed only to be Sufficient for End-user Needs

What was important was that the technology capabilities that were available were perceived by the participants as being sufficient for their needs. Examples of this included the bone marrow transplant teleconsultation project at W1 and X2’s tuberculosis teleconsultation project. In these cases, the radiographic images transmitted utilizing the teleconsultation equipment were not expected to be useful because these sites did not have a digital scanner or cameras with resolutions that met the standards set by the American College of Radiological Society for digital radiographic images. However, the specialists found the quality of the images transmitted using either the video camera focusing on a backlit image or a standard Elmo document camera were more than sufficient for their sensemaking needs. An oncologist involved in the bone marrow transplant project at HSC W described during the first data collection period:

*Looking at the (patient’s) X-rays directly over the [teleconsultation equipment] has been very helpful. They come through clearer than I ever imagined they could….It was critical to the consult(ation)—we are using a document camera to image CT-scans. And at my level of radiological sophistication, that is actually enough. It is actually a very nice picture, enough so that we can look at their CT-scans.*

In this case, relatively simple technology (a document camera) was more than sufficient for the end-users’ needs. Most likely this was because the teleconsultation project was being utilized for learning so it enabled the RSHCP to work with the HSC specialist to develop an understanding of what was happening and not as a tool for information processing where the HSC specialist was using the transmitted image to make a definitive diagnosis. For example, the infectious diseases specialist described during the first data collection period what happened when she had asked a radiologist for his reading of radiographic images transmitted via the teleconsultation equipment:

*I had the radiologist come over here and he said he would not be willing to give a formal, legal reading off of it but he could give what they sometimes call a wet (preliminary) reading. It would be similar to if they were doing an upper GI and they were watching a fluoroscopy, they would be watching it on a screen but not the printed sinofilm. So he was also able to make a wet reading, but legally, he felt uncomfortable reading films off of it.*

Indeed, the findings indicated that when teleconsultation projects were utilized for learning, having too sophisticated technology was often worse than having too little technology because it inhibited one or more of the parties from believing that the technology worked for them. This led to circumstances where participating healthcare providers believed that the technology didn’t work when it actually did. As one information systems professional at Site W explained during the first data collection period:

*If the physicians do not know how to use the equipment, or if they are afraid of the equipment, they say the equipment doesn’t work when actually the physicians don’t know how to use it.*

An excellent example of this occurred in Project ECDD, where the specialists at HSC Z did not believe they could fully control the remote site cameras in the newest generation of teleconsultation workstations (as they could in the prior generation of equipment) when in fact they actually could. This significantly impacted the perceived usefulness and appropriateness of the workstation because a fixed camera at the remote site was fine as long as the patient could sit still. Unfortunately, most of the patients were young children who, not surprisingly, were not always cooperative. HSC Z ECDD’s Director explained:

*(Y)ou can zoom in on a child’s face if they are in a wheelchair—they’re not running around the room. When you have a 2½ year old with Attention Deficit Disorder running around it doesn’t work. But a child in a wheelchair we can zoom in.*

## Discussion

The purpose of this research was to better understand how a complex adaptive system dominant logic of teleconsultation differs from a mechanistic dominant logic of teleconsultation, and to identify the implications for researchers and practitioners of applying a CAS dominant logic to evaluation and understanding of teleconsultation projects. Teleconsultation projects were analyzed from both a mechanistic and CAS dominant logic in order to further understand the influence of dominant logic on utilization rates of teleconsultation projects. This research also demonstrated why, contrary to generally accepted arguments, neither technology capabilities limitations nor limited reimbursement were likely responsible for the low utilization rates and lack of sustainability of effectively implemented teleconsultation projects.

In both dominant logics, the objective of teleconsultation projects was to increase access to and quality of healthcare delivery to medically underserved areas and populations in a cost efficient manner. A mechanistic dominant logic belief was that teleconsultation projects closely resembled the traditional service delivery model where generalists (RSHCPs) handed off the responsibility of their patients with difficult cases to HSC specialists, while a CAS dominant logic focused on the system’s emergent behavior of learning resulting from the relationships and interactions of participating healthcare providers. Implicit in the mechanistic dominant logic was the assumption that the primary focus of the projects would be increased information processing and exchange capabilities. For example, teleconsultation systems were designed based on the assumption that RSHCPs would utilize teleconsultations to provide HSC specialists with the information needed for a definitive diagnosis so that the HSC specialists would in effect take over providing patient care, in this case by communicating to the RSHCPs what needed to be done.

However, the findings indicated that the emergent behavior of effective and sustainable teleconsultation projects differed significantly from what was anticipated in a mechanistic dominant logic. Additional information processing capabilities were of limited value because rarely did RSHCPs have little or no idea about what was going on or what needed to be done, and the teleconsultation sessions very rarely were used for definitive diagnoses by HSC specialists. A mechanistic dominant logic has poor explanatory power in terms of the sustainable impact a teleconsultation project can have on remote site healthcare delivery because it was based on the incorrect or insufficient assumption that the RSHCPs in medically underserved environments were looking for more information in order to address the complexity they faced when it was sensemaking, learning, and reassurance that they wanted and needed. A CAS dominant logic, with its focus on emergent behavior and the importance of relationships and interactions between agents, explains why the emergent behavior of teleconsultation projects that were effective and sustainable was different than expected. A CAS dominant logic shifts the focus of teleconsultation projects from one of providing a simple service to one of learning. The systems goal becomes improving local capacity by leveraging HSC specialists’ expertise and empowering participating RSHCPs so that they could handle both a greater variety and more complex healthcare problems on their own.

Consistent with CAS dominant logic, the findings indicate that teleconsultation projects that continued to be utilized involved participants taking on new roles and continuously learning. The burn unit, Project ECDD and Project HCV teleconsultation projects were excellent examples of this. In contrast, those teleconsultation projects that followed a mechanistic dominant logic where traditional roles were maintained tended see utilization rates fall or cease altogether. Excellent examples of this included X1 pediatric oncology and Y2 multiple medical specialties teleconsultation projects.

The findings also suggest that the learning that occurred in teleconsultation projects had to be continuous in nature because it enabled RSHCPs to better handle the constantly changing nature of the problems faced; otherwise one-off training involving information exchange would have been sufficient. This is not to say that learning that primarily involved information exchange was not useful; rather, it could not be the primary teleconsultation project learning behavior.

### Teleconsultation Technology from a Mechanistic and CAS Dominant Logic

Technology plays a critical role in a mechanistic dominant logic of teleconsultation. Since teleconsultation is perceived as replicating the traditional face-to-face interaction in service delivery, teleconsultation technology must be quite sophisticated and is judged on its ability to replicate the face-to-face interaction. The literature is filled with statements declaring that the technology has been a major barrier to the widespread utilization of teleconsultations, and continues to suggest that very complex and sophisticated technology is needed for teleconsultation [1, 26]. Therefore, from a mechanistic dominant logic, technology issues must be addressed before teleconsultation projects can be expected to be widely utilized.

The problem is that there is almost no empirical evidence to support this belief. In fact, empirical evidence suggests that technology capabilities sufficient for teleconsultation projects to have a positive impact on remote site healthcare delivery have been available and deployed for nearly 20 years and have been utilized by RSHCPs with very limited training and support available [42]. Further, teleconsultation technology functionality, like all information and communications technology, has increased significantly, become easier to use, more widespread, and much more affordable during this time.

The most common technology barriers identified include a lack of broadband availability, cost, ease of use, and end-user familiarity [1, 26, 56]. However, in rural parts of the United States, the setting for this research, the availability and affordability of high-speed bandwidth has increased dramatically as a result of government initiatives [23, 26, 57]. Further, as technology costs in general have decreased, telemedicine technology costs have fallen as well [6]. For example, during the first data collection period, the typical cost for a high-speed telecommunication link, usually either T1 lines or satellites, tended to be up to $3500 per month. By the time of the second data collection period, the typical cost fell to $200 per month as shared IP-based multipoint telecommunication networks replaced T1 lines and satellites.

In terms of ease of use, even at the time of the second data collection period, connecting to a teleconsultation session had been simplified for the end user as the result of the introduction of graphical user interfaces. Many of the sites studied during the second data collection period utilized off-the-shelf videoconferencing equipment for their teleconsultation sessions, and the ease of use of such equipment has likely significantly increased since then. Further, similar to other telecommunication networks, the reliability and stability of the networks utilized in telemedicine have likely increased—reducing the need for technical support. Finally, end-user familiarity using similar technology to connect to and interact with the Internet, combined with the widespread adoption of smart phones, makes it likely that end-users are now much more comfortable utilizing information and communication technologies in general.

This and prior research has found that only limited technology capabilities are needed. The technology used and its usage in the teleconsultation projects studied was all relatively simple and straightforward. In other words, neither the technology nor the processes to which it was being applied were very complex relative to the technology available and the projects being attempted. The technology itself, as opposed to end-user familiarity with the technology and the training they received, was not perceived as an issue in either of this research’s two data collection periods, and the technology has evolved substantially since then. Therefore, it is unlikely that it is currently a barrier to teleconsultation sustainability.

A CAS dominant logic furthers our understanding of why this is the case and provides a theoretical framework by which to explain why the teleconsultation literature about the role of technology, which is based on a mechanistic dominate logic, does not have adequate explanatory power. A mechanistic dominant logic of teleconsultation following the traditional service delivery model implies that very sophisticated technology was required. In contrast, the findings indicated the effective practice of teleconsultation required only limited technology capabilities. This was consistent with a CAS dominate logic that the technology mattered only to the extent that the project participants found it to be sufficient for their needs. A CAS dominate logic of teleconsultation views technology as an independent agent whose characteristics are not judged by its sophistication or ease-of-use but by the extent to which other agents find it useful in what they wanted to do.

In part, relatively simple technology configurations were sufficient because the variability in the technology capabilities required by the different teleconsultation projects was actually quite limited. Projects HCV and DABC used the teleconsultation equipment primarily for conferences between healthcare providers, and a number of Project HCV remote sites used only teleconferencing to participate—which they found to be sufficient for their needs. Project ECDD’s technology capabilities requirements were greater than the other projects in that they also used the equipment for such things as patient evaluations based on patient movement or sounds made, and for demonstrating to the remote site developmental specialists how to manipulate patient body parts as part of an evaluation or course of therapy. However, these greater technology capabilities were not cutting edge. As previously discussed, what they mainly needed in terms of additional technology capabilities was the ability to fully control the remote site cameras. Therefore, even the more demanding technology requirements of Project ECDD were very limited in terms of the technology capabilities available.

### Reimbursement from a Mechanistic and CAS Dominant Logic

A CAS dominant logic of teleconsultation also explains why limited reimbursement was not perceived as a major barrier to project utilization. For the HSCs, there were a number of possible explanations for this. First, the amount of time that individual participating HSC specialists allocated to nonspecialty teleconsultation projects was quite limited and averaged approximately one session per month. Second, in the case of specialty teleconsultation projects involving conditions with long-term treatment regimens or follow-up, the HSCs often were reimbursed on a global fee basis—making teleconsultation session reimbursement a moot point. Third, many of the teleconsultation sessions involved indigent care, where the HSC specialists were not going to be reimbursed whether the patient was seen via teleconsultation or in the clinic. Moreover, the HSCs studied had not developed the administrative processes necessary to file reimbursement claims for eligible teleconsultation sessions. Finally, many of the teleconsultation project sessions were not eligible for remote site reimbursement because the patient was not present during the teleconsultation sessions themselves. As such, consistent with a CAS dominate logic, limited reimbursement did not have a significant impact on the relationships and interactions between the participating healthcare professionals, nor did it appear to be a significant characteristic of the environment to which teleconsultation projects had to adapt.

### Changes in Regulations

Since the second data collection period, a number of new regulations and laws potentially impacting teleconsultation utilization have been enacted. Key among these in the United States was the passing of the Patient Protection and Affordable Care Act of 2010 (ACA) [58]. ACA goals include increasing health insurance and healthcare availability and affordability for the uninsured and those with low incomes. The ACA encouraged the establishment of accountable care organizations and is in the process of instituting changes in reimbursement that encourage and reward physicians and organizations for the quality and efficiency of care instead of the number of services provided [58]. For example, the Centers for Medicare and Medicaid Services (CMS) is implementing and enhancing programs such as Comprehensive Primary Care Plus. This program rewards physicians for providing high quality and efficient care, and increases physician flexibility by allowing them to choose measures and activities appropriate for the type of care provided [59]. Further, the ACA specifically identifies telehealth as an innovative means by which to provide and coordinate care related to chronic conditions and behavioral health issues for medically underserved areas, and as a meaningful tool for accountable care organizations to provide high quality and efficient healthcare services in a cost effective manner [60].

A CAS dominant logic of teleconsultations is consistent with the ACA’s philosophy and goals. For example, a CAS dominant logic of teleconsultations focuses on the effectiveness of such projects in providing healthcare and not the extent to which a teleconsultation session resembles a face-to-face session. Further, many of the types of teleconsultation projects studied in this research that were sustained over time were consistent with the role for telehealth specified by the ACA in that they focused on chronic conditions or conditions that had long treatment and follow-up regimens. An important aspect of these projects was their flexibility in terms of the roles that both the HSC specialists and RSHCPs filled. This enabled them to engage in activities appropriate for the type of care provided. Therefore, it appears that a CAS dominant logic of teleconsultations is consistent with the goals and philosophy of the ACA.

It also can be argued that, consistent with a mechanistic dominant logic, changes related to reimbursement could be a driving force behind increased teleconsultation utilization rates and sustainability in the near future. By 2015, 48 states had approved some type of reimbursement for services provided by telehealth [61], and the CMS has and continues to test changes in its reimbursement model so that teleconsultations and other telehealth activities are reimbursable [62]. What the CMS does is often adopted by private insurance plans. For example, Blue Shield/Blue Cross, a major private insurer, now covers 24/7 teleconsultation services in many of its health insurance plans [63].

However, these changes related to reimbursement, while helpful, actually may not be significant factors influencing the future of teleconsultations. For example, changes in reimbursement regulations and policies since the second data collection period would not have changed the number of teleconsultation projects that qualified for reimbursement (regulations had already changed so physicians were no longer required on both ends). Of the fourteen teleconsultation projects still in existence at the time of the second data collection period, at most five of the teleconsultation projects’ sessions (Y3—Burn Unit, Y4—Oncology, and Z5, Z6, and Z7—ECDD) would be reimbursable all the time while only three (Y1—Multiple Medical Specialties, Y6—Pediatric Care, and Z8—DABC) would be reimbursable at least part of the time. Five of the remaining teleconsultation projects (X2—Multiple Drug-Resistant TB and Z1-Z4—Hepatitis C) were not and would continue not be eligible for reimbursement because they did not meet the requirement of a patient being present during the session. The remaining teleconsultation project (Y5—Primary Care) was not eligible for reimbursement because an emergency medical technician was the RSHCP. Further, reimbursement in Y3—Burn Unit would be limited to only the remote site facility because the HSC was paid on a capitated basis. Thus, the impact of changes in reimbursement regulation were likely to be limited in terms of teleconsultation product utilization and sustainability.

### Contributions to Research and Practice

This research contributes to both research and practice by providing an alternate conceptualization of the dominant logics of teleconsultation that furthers understanding of how project utilization rates can be improved. We suggest that attention must be paid to the dominant logic driving the system. Considering a CAS dominant logic changed the focus from the intended behavior of the system to its emergent behavior. This research also highlights the importance of the interactions and relationships among the teleconsultation project participants. This conceptualization can be utilized by practitioners to both evaluate potential, planned, and implemented teleconsultation projects, and provide useful prescriptions to improve the utilization and sustainability of existing teleconsultation projects.

### Limitations

This research is not without its limitations. First, even though drawing on data collected at two different points in time, this research was not actually multiperiod because much of the data included cases that were not active at the time of the first data collection period. However, it can be argued that in some ways this further strengthens the findings presented because inferences could be drawn from data about projects that were relatively inactive or not sustained, and these inferences could be compared against the characteristics of those teleconsultation projects that were sustained over time. It is argued the timing of the two data collection periods was appropriate and enabled the collection of the necessary data. While there are many reasons for this, a key reason was that most teleconsultation projects at the time of the first data collection period started as pilot studies or proof of concept, while those from the second data collection period occurred after the efficacy and efficiency of teleconsultation for many clinical activities had been established and the deployed teleconsultation projects were now being done as part of organizations’ ongoing operations.

Second, this research involved only teleconsultation projects located in the United States, which has its own characteristics in terms of healthcare providers, payers, and regulations which may not hold in other parts of the world. This research needs to be replicated in additional countries with differing healthcare systems.

Third, while the sample size was limited, it is argued that the diversity in the types of healthcare activities practiced, the professional qualifications of healthcare providers involved, and population size, location, and remoteness of the sites themselves makes this an appropriate sample. The results between those teleconsultation projects located in areas designated metropolitan and those in nonmetropolitan areas exhibited no meaningful difference. The majority of remote sites in this research were located in nonmetropolitan areas and were in effect rural. Rural areas tend to face healthcare challenges that are similar to or in some cases more pronounced than urban areas because rural populations tend to be poorer, older, and have higher rates of certain chronic diseases [1, 64, 65].

### Future Research

In addition to addressing the limitations discussed above, future research needs to examine what and how differences in relationships impact the utilization and sustainability of teleconsultation projects. How the characteristics of such relationships differ in terms of factors that enable emergent behavior also needs to be studied. Further, how the key factors that facilitate and inhibit relationships’ emergent behavior need to be identified and understood.

## Conclusion

This research has examined teleconsultation projects utilizing both a mechanistic and CAS dominant logic. When a project is designed with a mechanistic dominant logic, it is less likely to be sustained, whereas a project designed with a CAS dominant logic is more likely to be sustained. A CAS dominant logic focuses on the emergent behavior resulting from the interaction and relationships among the participating healthcare providers and provides a better understanding of how and why some teleconsultation projects differ in terms of their health care delivery impact and sustainability.

## Appendix

### Sample Interview Questions

#### Ongoing Telemedicine Projects and Impact

- Describe the ongoing telemedicine projects currently at your site.
- Which of the projects would you describe as being the most successful? Why?
- Which of the projects would describe as being the least successful? Why?
- Can you give some examples of the telemedicine activity being used?
- What happened or would have happened prior to the advent of telemedicine? (examples)
- How do you think telemedicine has changed the way things are done? (examples)
- What are some of the benefits you see? (examples)
- What are some of the things you are disappointed with or dislike about telemedicine? (examples)
- How often are the telemedicine sessions held?
- What has patient reaction been like?
- How do you feel it has impacted the patients?
- How would you go about this project differently if you could?
- How do you see this project evolving?
- What role do you see telemedicine playing at this institution?
- What lessons have you learned?

#### Information Technology Configuration

- Describe your telemedicine technology setup. (get demonstration if possible)
- What equipment do you have here/there?
- What is the network configuration?
- Have you had any problems with the equipment? (examples)
- What happens if the system breaks here/there/network? (examples)
- What generation of equipment is this?
- What is your opinion of the system in terms of functionality/reliability/ease of use? (examples)
- What do you like/don’t like about the system? (examples)
- Who operates the equipment here and there?
- What kind of training do they have/did you get?
- How much does the network connection cost?
- Who funded the purchase of this equipment?
- What are your future IT plans?

#### Key Informant Involvement in Telemedicine

- How and why did you get involved in telemedicine program in general?
- How and why did you get involved in this specific telemedicine project?
- Who was the champion or prime motivator for this project or program in general?
- What do you see as the objective of this project?
- What is your involvement with the telemedicine program?
- Are you compensated for participating in the telemedicine project?
- Has it made doing your job more difficult or easier?

#### Funding

- Where did the funding for this project come from?
- When does this funding end?
- What are you going to do then?
- What do you believe the impact will be if this project is not renewed?

## Additional file

Additional file 1: Table S1. Teleconsultation projects background and demographics. (XLSX 16 kb)

## Abbreviations

ACA: Patient Protection and Affordable Care Act of 2010
CMS: Centers for Medicare and Medicaid Services
DABC: Drug abuse and behavioral counseling project
ECDD: Early childhood developmental disabilities project
HCV: Hepatitis C virus project
HIPAA: Health Insurance Portability and Accountability Act of 1996
HPSA: Health professional shortage area
HSC: Health sciences center
IT: Information technology
RSHCP: Remote site healthcare provider

## Footnotes

1 The one exception was a pediatric oncology teleconsultation project that was discontinued by the pediatric oncologists after a period of 4 months.
